# Recovery from heat‐induced infertility—A study of reproductive tissue responses and fitness consequences in male *Drosophila melanogaster*

**DOI:** 10.1002/ece3.9563

**Published:** 2022-11-30

**Authors:** Berta Canal Domenech, Claudia Fricke

**Affiliations:** ^1^ Institute for Evolution and Biodiversity University of Muenster Muenster Germany; ^2^ Muenster Graduate School of Evolution University of Muenster Muenster Germany; ^3^ Institute for Zoology Halle‐Wittenberg University Halle (Saale) Germany

**Keywords:** accessory glands, climate change, seminal fluid proteins, spermatogenesis, sterility

## Abstract

The predicted temperature increase caused by climate change is a threat to biodiversity. Across animal taxa, male reproduction is often sensitive to elevated temperatures leading to fertility loss, and in more adverse scenarios, this can result in sterility when males reach their upper thermal fertility limit. Here, we investigate temperature‐induced changes in reproductive tissues, fertility reduction, sterility, and the associated fitness loss during the subsequent recovery phase in male *Drosophila melanogaster*. We heat‐stressed males during development and either allowed them to recover or not in early adulthood while measuring several determinants of male reproductive success. We found significant differences in recovery rate, organ sizes, sperm production, and other key reproductive traits among males from our different temperature treatments. Sperm maturation was impaired before reaching the upper thermal sterility threshold. While some effects were reversible, this did not compensate for the fitness loss due to damage imposed during development. Surprisingly, developmental heat stress was damaging to accessory gland growth, and female post‐mating responses mediated by seminal fluid proteins were impaired regardless of the possibility of recovery. We suggest that sub‐lethal thermal sterility and the subsequent fertility reduction are caused by a combination of inefficient functionality of both the accessory gland and testes.

## Graphical abstract

We investigate the consequences of heat stress and the mechanisms of heat‐induced fertility loss and sterility in *Drosophila*.

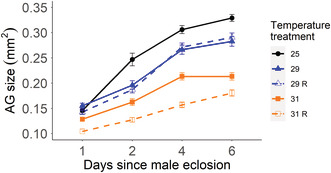

## 1 INTRODUCTION

Temperature is a critical abiotic factor for many organisms and can turn into an environmental stressor, particularly for ectotherms. Elevated temperatures are known to affect an ectotherm's physiology, behavior, and, on a broader scale, its overall performance (Huey & Stevenson, 1979). When critical thermal limits are exceeded, both viability and reproductive potential are harmed (Sales et al., 2021). In recent years, most research assessing phenotypic effects under increasing temperatures has incorporated mainly measures that evaluate an organism's physiological failure (viability thresholds), like death or heat coma (Walsh et al., 2019). However, often the fertility range is narrower than the viability range, and thus, fertility limits can be reached before the onset of mortality, such that sub‐lethal temperatures already pose an important fitness loss impeding organisms to reproduce, threatening population stability, and persistence (Kellermann et al., 2012; van Heerwaarden & Sgrò, 2021; Walsh et al., 2019). Given the strong ecological implications that fertility loss and sterility exert on organisms, studying the capacity of species and the mechanisms to respond ecologically and evolutionarily to the challenges of increasing temperatures has become an important research area, especially within the climate change context (David et al., 2005; Parmesan, 2006; Parratt et al., 2021; Sales et al., 2021; Walsh et al., 2019).

Sterility is one consequence when reaching thermal fertility limits at both the upper and lower ends, for example, via exceedingly extreme temperature exposures. Most of the literature refers to males, as these have repeatedly been found to be more temperature sensitive than females (e.g., Sales et al., 2018; Zwoinska et al., 2020). However, there are also examples of female sterility due to high temperatures (e.g., in the Nile tilapia, *Oreochromis niloticus* [Byerly et al., 2005]). Based on the premise that spermatogenesis is more thermosensitive than oogenesis, the study of male fertility thresholds is of special interest, not only to understand the damage imposed on male fertility but also to gauge the consequences for reproductive capacity and thus, fitness (Parratt et al., 2021). Even though this phenomenon has been documented for a range of taxa‐like insects (e.g., several *Drosophila* species such as *D. melanogaster* and *D. simulans* [Chakir et al., 2002]), fishes (e.g., Nile tilapia, *O. niloticus* [Byerly et al., 2005], and channel catfish, *Ictalurus punctatus* [Strüssmann et al., 1998]), reptiles (e.g., yucca night lizard, *Xantusia vigilis* [Cowles & Burleson, 1945]), and some vertebrates (e.g., Arbor Acres roosters, *Gallus gallus* [McDaniel et al., 1996], zebra finch, *Taeniopygia guttata* [Hurley et al., 2018], and rams, *Ovis aries* [Hafez, 1964]), the mechanisms underlying sterility at extreme temperatures are still unknown. The fact though that some species can recover fertility after being transferred to milder temperatures (e.g., *D. melanogaster* males [Chakir et al., 2002]) indicates that the destruction of germ cells might not explain the observed temperature‐induced infertility.

Previous research on *D. melanogaster* has shown that elevated temperatures disrupt spermatogenesis causing cytological abnormalities (Rohmer et al., 2004); for example, males had shorter cysts, the shape or position of sperm nuclei was altered, spermatids did not elongate properly, and an increase in spermatid death rate was observed. *D. simulans* similarly had shorter cysts at higher temperatures (David et al., 2005). These changes are not necessarily always negative, for example, temperature‐dependent plasticity in sperm length was shown to be adaptive in *Tribolium castaneum* (Vasudeva et al., 2019). Despite these first studies on sperm morphology under thermal stress, little is yet known about recovery dynamics on sperm production (but see Sales et al., 2021). Furthermore, effects on other key reproductive tissues such as the male accessory glands (AGs) have not been considered. The importance of AGs for male reproductive success in *D. melanogaster* has been widely studied (Chen, 1984; Wigby et al., 2020; Wolfner, 1997). Seminal fluid proteins (SFPs) secreted mainly by the AGs are transferred together with sperm to the female during mating causing changes in female post‐mating responses (e.g., behavior and physiology [Chapman, 2001; Chen et al., 1988]). In addition, SFPs affect male sperm competitiveness and modulate sperm storage dynamics inside the female's sperm storage organs (Avila et al., 2011), together ensuring fertility. Moreover, SFPs might have protective functions as in honeybees, *Apis mellifera* (den Boer et al., 2009), and leaf‐cutter ants, *Atta colombica* (den Boer et al., 2007), where SFPs increase sperm viability. Hence, whether temperature damages either or both tissues need to be considered to understand the mechanisms of temperature‐induced sterility.

With the predicted temperature increase (at least 1.5–2°C for 2081–2100, Collins et al., 2013) and the occurrence of longer and more severe heat waves (Collins et al., 2020; Meehl & Tebaldi, 2004) due to global climate change, we think that studying the responses of reproductive traits to stressful thermal conditions is of special interest in order to determine species persistence under possible new environmental conditions (Hoffmann, 2010; Huey & Kingsolver, 1993; Huey & Stevenson, 1979; Kellermann et al., 2012; Sinclair et al., 2016; van Heerwaarden & Sgrò, 2021; Walsh et al., 2019).

In this context, we assessed fitness loss and the ability to recover, focusing on the mechanistic basis of heat‐induced sterility, of males exposed to sub‐lethal developmental temperatures. Life stages undergoing fundamental changes might be particularly sensitive to environmental stressors (Lowe et al., 2021), for example, the pupal stage in the oriental fruit moth, *Grapholita molesta* (Zheng et al., 2017) or *Drosophila* larvae (Hoffmann et al., 2003). In addition, the lack of mobility of many species at both early and late developmental stages adds a challenge to elude thermal stress. Hence, we here exposed larvae to heat stress and considered the resulting consequences on male fertility in early adulthood. To determine the causes of male temperature‐induced sterility, we tested whether spermatogenesis is disrupted impairing mature sperm formation, and secondly, measured whether a delay in AG maturation (Ruhmann et al., 2016) contributes to reduced reproductive success. With this extensive analysis of male reproductive traits, we suggest that impaired functionality of both reproductive tissues is causing temperature‐induced male sterility.

## 2 MATERIALS AND METHODS

### 2.1 Fly stocks and culturing

We used a *Drosophila melanogaster* wild‐type stock collected in Portugal by Prof. Élio Sucena in Azeitão, in 2007. It was established as an outbred population from 160 wild‐caught fertilized females with an ample degree of genetic variation within the population (Martins et al., 2014). Flies were cultured in our laboratory at standard conditions: 25°C and 60% humidity at a 12 h light–dark cycle. Stocks were kept in glass bottles filled with 70 ml of standard yeast–sugar (SYA) food (Bass et al., 2007). Once a week, three glass bottles with about 250 recently eclosed flies each were started, and we mixed flies across bottles regularly to maintain genetic diversity thus, overlap between generations could occur. We used a temperate population as we expected them to maintain higher phenotypic variation; they were initially adapted to exist within a broad thermal range compared with tropical populations (Hoffmann et al., 2003). Species from temperate areas are expected to maintain higher phenotypic plasticity even after adaptation to laboratory conditions and provide a more promising way to test thermal responses under a broader range of experimental temperatures and hence, give a more powerful estimation of a species' ability to cope with increasing temperatures (Mathur & Schmidt, 2017). Although laboratory adaptation may alter some life‐history traits, previous research has shown that some plastic responses are maintained (Trotta et al., 2006) and previous research in thermal responses still finds ample variation (e.g., Parratt et al., 2021; Sales et al., 2018).

For paternity analysis in a sperm competition experiment, we used flies bearing the stubble (*Sb*) mutation as a tractable phenotypic marker. The *Sb* gene was back‐crossed multiple times into the wild‐type Dahomey genetic background. *Sb* is a dominant mutation that causes a short, thick bristle phenotype (Lees et al., 1945) that is visible by the eye and can be easily distinguished from the wild‐type bristle structure. As the recessive homozygous phenotype is lethal, we used heterozygous males in the subsequent sperm competition assay. The stock was kept under the same standard conditions as described above.

Throughout all assays, in order to obtain experimental flies, we allowed parental flies to mate for 24 h and oviposit on grape juice agar plates (50 g agar, 600 ml red grape juice, 42.5 ml Nipagin [10% w/v solution] and 1.1 L water) with a semi‐liquid baker's yeast paste distributed all around the plate to promote egg laying. We incubated plates for 24 h and collected the first instar larvae at a density of 100 larvae per vial (95.0 mm in height × 25.0 mm in diameter) containing 7 ml of SYA food. For all experiments, flies were collected within 8 h after eclosion as virgins on ice. Adult flies were kept in separate sex groups of 20 per vial. Throughout the experiments, females were grown at 25°C, while males were exposed to different temperature treatments during development. We first tested how developmental temperature affects male fertility and whether males can recover fertility when placed at a benign temperature after eclosion. Control males were raised at the standard temperature of 25°C. We choose the most appropriate timeline and temperature treatments for further experiments based on previous data on the same species (Chakir et al., 2002). In this example, Chakir et al. (2002), measured fertility and fecundity for 10 days in males developed at temperatures ranging from 29 to 31°C, at 1°C increments. They found that both fertility and offspring output recovered and remained constant from day 6 to 10, for males previously exposed to temperatures of 29°C or above. This trend remained below the control group throughout the experiment, indicating that major changes in fertility occurred at an earlier age (Chakir et al., 2002). Thus, based on these data, we chose to focus on changes in fertility from day 1 to 6, and at temperatures above 28°C. In this way, we exposed males to two stressful temperatures, one severe (31°C) heat stress (near to the lethal threshold for *D. melanogaster* of 32°C; Petavy et al., 2001) and one moderate stress (29°C). As we were interested in the fitness consequences of the recovery process and the underlying mechanisms, we allowed half of the males to recover from the heat stress (denoted with an R) after eclosion, while keeping the other half at the stressful temperature. All assays described below were done under these conditions. In order to maintain temperatures precisely for our different treatments, incubators with ±0.5°C accuracy were used (INCU‐Line® IL 10). Accuracy was monitored by placing a temperature logger (NOVUS®; accuracy of ±0.5°C) in each incubator throughout the course of the experiments to record the temperature.

### 2.2 Fecundity experiment

We measured egg‐laying rate, fertility, fecundity, and egg‐to‐adult survival of females once mated with a temperature‐stressed male who was either allowed to recover or not. Mating assays took place on 3 different days depending on male age: 2, 4, and 6 days after eclosion, in order to infer changes in fertility during sexual maturation. On each assay day, virgin males of the appropriate age were paired with 5‐day‐old virgin females, with 40 pairs per treatment and day initially set up. Pairs were observed by three people for 7 h on days 2 and 4, and for 5 h on day 6. The observation duration was adjusted to reach an appropriate sample size for all treatments (see Table 1 for final sample sizes). Each pair was allowed a single mating, and males were discarded after a successful mating. Pairs that did not mate within the observation time were discarded. Mated females were kept at 25°C until the next day allowing them to lay eggs until the mid‐point of the observation period and were then discarded. Two people counted the number of eggs and the eclosed offspring after the vacated vials were kept at standard conditions for 12 days. Both observers counted a subset of vials conjointly to ensure that egg counts matched before counts were recorded. From the number of offspring counted, we estimated the egg‐to‐adult survival by subtracting the number of eclosed adults (success) from the initial number of eggs (to gain the number of fails). We then analyzed the proportion of successful versus failed events with a binomial model. This assay was repeated independently following the same procedure, but we added one more mating point shortly after eclosion and thus measured male fecundity for days 1, 2, 4, and 6. With this addition, we got a better understanding of the effects of heat stress on fertility and fecundity in recently eclosed males.

**TABLE 1:** Sample sizes attained in the sperm competition (left) and fecundity (right) experiments

Sperm competition experiment				Fecundity measures on day		
Treatment (°C)	*N*_0_	Mated	Remated	2	4	6
25	50	37	13	33	33	37
29	70	44	44	22	32	33
29 R	70	37	37	19	31	34
31	70	9	9	8	32	41
31 R	70	19	19	15	13	16

*Note*: The left panel shows mating success of first and second matings for the sperm competition assay: Number of pairs set up initially (*N*_0_), number of successfully mated pairs after the first mating opportunity, and number of successfully remated pairs gained during the second mating opportunity 48 h after the initial mating. Focal males used during the first mating opportunity were grown at 25, 29, or 31°C and kept at the growth temperature after eclosion or moved to the control temperature to recover (R). *Sb* competitor males grown at 25°C were used in the second mating opportunity. In the right panel, males were allowed a single mating at 2, 4, or 6 days after eclosion when developed at the same temperatures as above.

### 2.3 Remating and sperm competition experiment

To further examine determinants of male reproductive success, we assessed male post‐mating competitive ability. We tested male sperm defense ability by first mating females to a temperature‐stressed focal male and subsequently, to an *Sb* mutant competitor. In this assay, all males and females were 5 days post‐eclosion when mated. Females and the *Sb* competitor males were grown at 25°C. For the first mating with the focal males, 50 (for the 25°C treatment) or 70 (for the 29 and 31°C treatments) individual pairs were set up and given 3.5 h to mate. We (two observers) continuously observed pairs and noted the time when pairs were set together, started, and ended mating to calculate mating latency and copulation duration. After successful mating, males were discarded, and females were allowed to lay eggs for 48 h. After this time, females were transferred to a new vial containing a virgin heterozygous *Sb* male. Pairs were allowed to mate for 2 h (see Table 1 for the total number of successfully mated pairs from both mating opportunities). We again recorded mating behavior as before and scored how many females remated. Females who remated were kept in the same vial for 2 days, allowing them to lay eggs, while *Sb* males were discarded. After these 48 h, we transferred females to new vials allowing them to lay eggs for 2 more days. We kept vacated vials from both the intermating interval and the 4 days after remating at standard conditions for 12 days allowing all the offspring to eclose. Vials were frozen and the offspring counted, whereby for the vials after remating, we determined paternity by separately counting offspring scored as presenting the *Sb* mutation versus the wild‐type phenotype. As Sb fathers were heterozygous for the mutation, and thus produced offspring with the *Sb* phenotype (those inheriting the dominant *Sb* allele) as well as the wild‐type phenotype, we corrected the counts for paternity scores. Assuming half the offspring fathered by the *Sb* males present, the *Sb* phenotype, and other half of the wild‐type phenotype, we doubled the *Sb* counts and corrected the wild‐type counts by subtracting the number of *Sb* offspring counted from the total wild‐type progeny number. The remaining difference in wild‐type counts was assigned as progeny to our focal males.

### 2.4 Maturation of the male reproductive system during recovery

In order to gain insights into the mechanistic basis of the recovery process, we measured changes in male accessory gland (AG) size during the first 6 days of recovery. We further checked for the presence of sperm in the seminal vesicles (SVs) and measured SV size and sperm viability to test the effects of elevated developmental temperatures on sperm production and quality. Under optimal conditions, 2‐ to 5‐day‐old males are expected to display mature sperm. By measuring SV size and the occurrence of sperm at different male ages, we can infer sperm maturation dynamics (Sitaram et al., 2014). All observations were done under a microscope (ZEISS, AxioVision Software) with an Olympus SC50 5‐megapixel color microscope camera at a 50X amplification. Further measurements (e.g., organ areas, wing length, and sperm head count) were carried out with ImageJ (Wayne Rasband).

#### 2.4.1 Accessory gland size changes during recovery

Measurements were done on glands dissected from virgin males, as a previous mating and the accompanying transfer of seminal fluid proteins reduces AG size (Koppik et al., 2018). We dissected glands from males 20–24 h‐, 2‐, 4‐, and 6‐day‐old and followed procedures as carried out by Ruhmann et al. (2016). We also measured wing length as an indicator of male body size to control for the allometric relationship between both factors (Shingleton et al., 2007). We measured both AGs and wings for each male and the mean was used for the analysis. In total, the AGs and wings of 15–26 males per treatment and day were measured.

#### 2.4.2 Seminal vesicle size and sperm presence

Measurements were done on 2‐ and 6‐day‐old virgin males. After dissecting the reproductive tract, we isolated both seminal vesicles (SVs). We placed the SVs on a slide in a drop of phosphate‐buffered saline (PBS) solution and a picture of both organs was taken under the microscope immediately after dissection to subsequently measure their area. Afterward, we checked for the presence of sperm by puncturing the central area with a thin needle. We recorded and assigned a score of 1‐ when sperm was present or 0‐ when the sperm was absent for each SV. When a male had one SV scoring 0‐ and one 1‐, then we overall assigned a score of 1‐ to that male. For SV size, the mean size of both SVs was used for each male in the subsequent analysis. A total of 15–20 males per treatment and day were dissected.

#### 2.4.3 Sperm viability

Measurements were done only in 6‐day‐old virgin males from the controls (25°C) and the 29°C recovery treatment, as especially young males and males from all the other treatments had a low number of sperm in the SV preventing reliable estimates. The experiment was done by two people blind to sample treatment, and daily measurements combining randomly samples from the two different temperature treatments were taken (Holman, 2009a). First, we dissected the male reproductive tract, and the SVs were isolated following the procedure described above. For sperm staining, we used a protocol specifically designed for *D. melanogaster* (Eckel et al., 2017) and adjusted the methodology of sperm extraction to avoid killing the sperm by mechanical manipulation. We gently squeezed the SV membrane making a “natural” tissue break without directly touching the sperm. We stained sperm with SYBR14® (1:50 in DMSO) and PI (LIVE/DEAD® Sperm Viability Kit, Thermo Fisher Scientific) following the protocol outlined in Eckel et al. (2017). The temporal decrease in sperm viability of the same male was measured at three different time points: just after the staining (t0), 15 (t15), and 30 (t30) min later. With this procedure, we could assess sperm quality and future sperm performance (Eckel et al., 2017). The time between staining and taking the first image was approximately 1 min. Pictures were taken under fluorescence (see microscope specifications above) by choosing randomly a square on the slide, done by a coworker unaware of the experimental design (Holman, 2009a). Pictures were taken in a dark room to prevent light damage and samples were additionally covered after staining. Sperm heads on pictures were counted by eye twice: once by an observer blind and by a second observer non‐blind to the treatment codes. As the counts were not significantly different, the mean of both counts was used for the data analysis. Green sperm were considered alive, whereas red and red–green double‐stained sperm were scored as dead. A total of 21 males per temperature treatment were used.

### 2.5 Data analysis

All tests were done using R (version 3.6.1) and RStudio (1.2.1335). Graphs were created with the “ggplot2” package in all cases (Wickham, 2016). As the data were not normally distributed, generalized linear mixed models (GLMM) were used with the appropriate error structure for analyzing the data for fecundity and sperm viability. In the first case, as the data suffered from an excess of zero counts, we used zero‐inflation models as advised by Zuur et al. (2009). We modeled both the likelihood of sterile replicates as well as factors explaining the number of offspring, combining both binomial and count part (with a negative binomial distribution) in one model; this needed packages “pscl” (Jackman, 2017), “lmtest” (Zeileis & Hothorn, 2002), and “glmmTMB” (Brooks et al., 2017). The models contained the following factors: temperature, day, and day^2^, as well as the interactions between temperature and day and temperature x day^2^. For the sperm viability analysis, we accounted for repeated measures of sperm from the same male across the three time points. In this case, time point and temperature were included in the model as fixed factors as well as the interaction between time point and temperature in the full model.

Fertility, male organ size (wing length, AG, and SV size), egg‐to‐adult survival, as well as mating behavior in the sperm competition experiment were analyzed with generalized linear models (glm) with the appropriate error structure and correction for overdispersion using the quasi‐extension when appropriate. The significance of factors was tested through an analysis of deviance by subtracting a factor from the full model and tested with an *F*‐ or Chi‐square distribution as appropriate for the error structure (Crawley, 2007). We only show significant factors retained in the final models. Most of the statistical analyses were done in two different ways: in the first case, all five treatments (developmental temperature and opportunity to recover or not) were considered separately by coding them as five different treatments. In the second approach, we instead included larval temperature (29 or 31°C) and recovery (yes/no) as two independent factors with a full factorial design. This approach, however, precluded us from using data from control males, allowing comparisons only among heat‐stressed males. As control males were not stressed and were exposed to a single temperature (25°C) during both development and adulthood, it was not possible to code this treatment as a binary recovery factor. In the main text, we always report the first approach unless the contrary is specified.

A Chi‐square test was applied to analyze sperm presence in the SVs and the mating and remating rates in the sperm competition experiment. Allometry between AG size and wing length was tested by using regression. For that, both variables were converted into the same units (μm^2^) and the data were transformed to a log scale for the analysis (Shingleton et al., 2007). Day was included as a fixed factor in the model to account for ontogenetic allometry through time.

Package “multcomp” (Hothorn et al., 2008) was used for the post hoc comparison of wing length. Pairwise comparisons using *t*‐tests were used for analyzing differences between temperature treatments in the AG size.

## 3 RESULTS

### 3.1 Sub‐lethal temperature effects on behavior and male reproductive output

**FIGURE 1:**
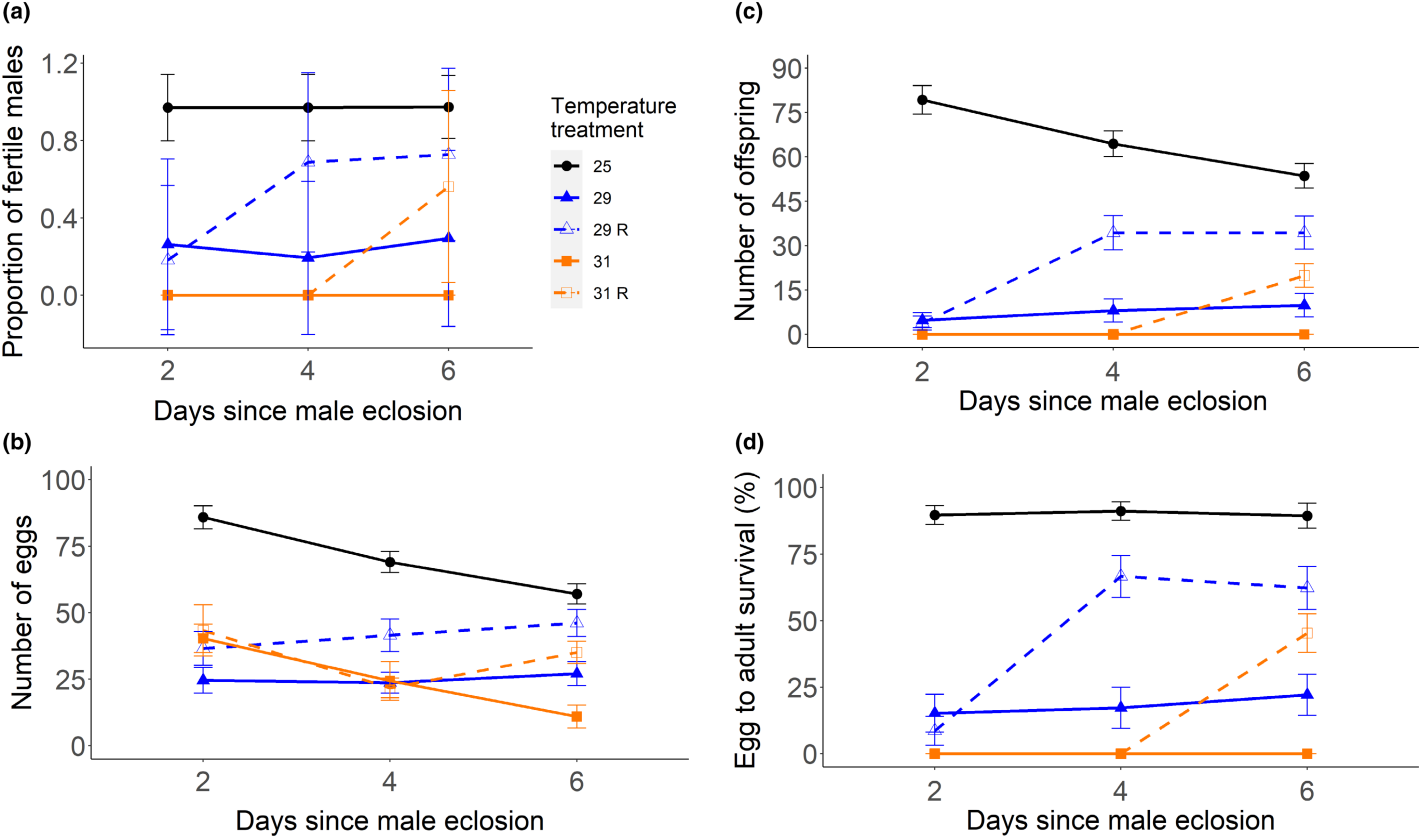
Comparison of reproductive output for heat‐stressed males and those allowed to recover (mean ± SE). The proportion of fertile males measured as the fraction of males at least producing one offspring (a), the number of eggs laid after a single mating (b), the offspring number (c), and the egg‐to‐adult survival (d). Males were allowed a single mating 2, 4, or 6 days after eclosion after having developed at (°C): 25 (circle symbol), black; 29 (triangle symbol), blue; or 31 (square symbol), orange. Males grown and kept after eclosion at the developmental temperature are shown as a solid line, while males allowed to recover (R) at 25°C after eclosion are shown as a dashed line.

On the basis that males of temperate *Drosophila melanogaster* strains become sterile at 30°C and the lethal threshold is reached at 32°C (Chakir et al., 2002; Petavy et al., 2001), we investigated the recovery dynamics for males of a temperate Portuguese strain (Martins et al., 2014) who developed at either moderately hot (29°C) or sub‐lethal (31°C) temperatures. First, we documented the viability of larvae under the different developmental temperatures and found those to be clearly stressful as only 45.5% respective 23.7% of flies eclosed when exposed to 29 or 31°C, in contrast to a 95% successful eclosion rate at 25°C. Remaining at 29 or 31°C was stressful to adults as well, as males clearly did not recover fertility in contrast to males allowed to recover (Figure 1a,c and Table 2).

**TABLE 2:** A generalized mixed model following a Gaussian distribution was used to analyze the number of eggs, and one with a quasibinomial distribution for egg‐to‐adult survival.

Factor	Deviance	*F*	*df*	*p*
Number of eggs				
Temperature	66,149	33.532	4	<.0001
Day	4804.2	4.871	2	.008
Temperature × Day	11,029	2.935	8	.004
Egg‐to‐adult survival				
Temperature	8607.6	56.138	4	<.0001
Day	1172.3	15.291	2	<.0001
Temperature × Day	1371	6.863	8	<.0001

Factor	Count part			Logit part		
	*df*	*χ* ^2^	*p*	*df*	*χ* ^2^	*p*
Offspring number						
Temperature	4	26.31	<.0001	12	162.81	<.0001
Day	2	0.23	.89	10	67.82	<.0001
Temperature × Day	‐	‐	‐	8	44.75	<.0001

*Note*: The offspring number produced was analyzed using a zero‐inflated negative binomial model. The model included a logit part that analyzed whether an offspring was produced or not, while the count part analyzed the number of offspring produced following a negative binomial distribution. Males previously developed at 25, 29, or 31°C and were kept at the growth temperature or moved to the control temperature to recover. Pairs were allowed to mate on days 2, 4, or 6 after eclosion. In all cases, male developmental temperature and opportunity to recover were analyzed as the factor temperature with five levels, with day of measurement coded as a factor with three levels.

We next assessed the fecundity of heat‐stressed males and found females mated once to one of those males laid significantly fewer eggs compared to females mated to control males (Figure 1b, Table 2). Males kept at stressful temperatures after eclosion induced lower numbers of eggs laid at each age tested, while a small improvement was seen for males allowed to recover for 6 days. We independently repeated this experiment with the addition of sampling also day 1 after eclosion and found the pattern to be robust (see Figure S1A,B and Table S1).

The offspring counts revealed that recovering males were initially sterile and recovered at different rates (see Figure 1c, interaction term Table 2), with offspring numbers approaching those of control males on day 6. This is matched by a steep increase in egg‐to‐adult survival between days 2 and 4, with males remaining in stressful conditions producing very few offspring and displaying a low egg‐to‐adult survival (Figure 1d, Table 2). No adult flies were produced by males grown and kept at 31°C. Males grown and kept at 29°C produced on average of 82% fewer offspring on day 6 with respect to control males, while those allowed to recover after eclosion had 36% fewer offspring, which equated to a 46% improvement in fertility. For males who developed at 31°C, we observed a slight increase of 37% in male fertility for those allowed to recover versus those not, but this modest increase in comparison to the control still signifies a 63% decline in offspring produced. Hence, at 6° above the optimum, males suffer a severe fitness loss even if allowed to recover, with persistent heat stress resulting in near‐complete sterility.

**FIGURE 2:**
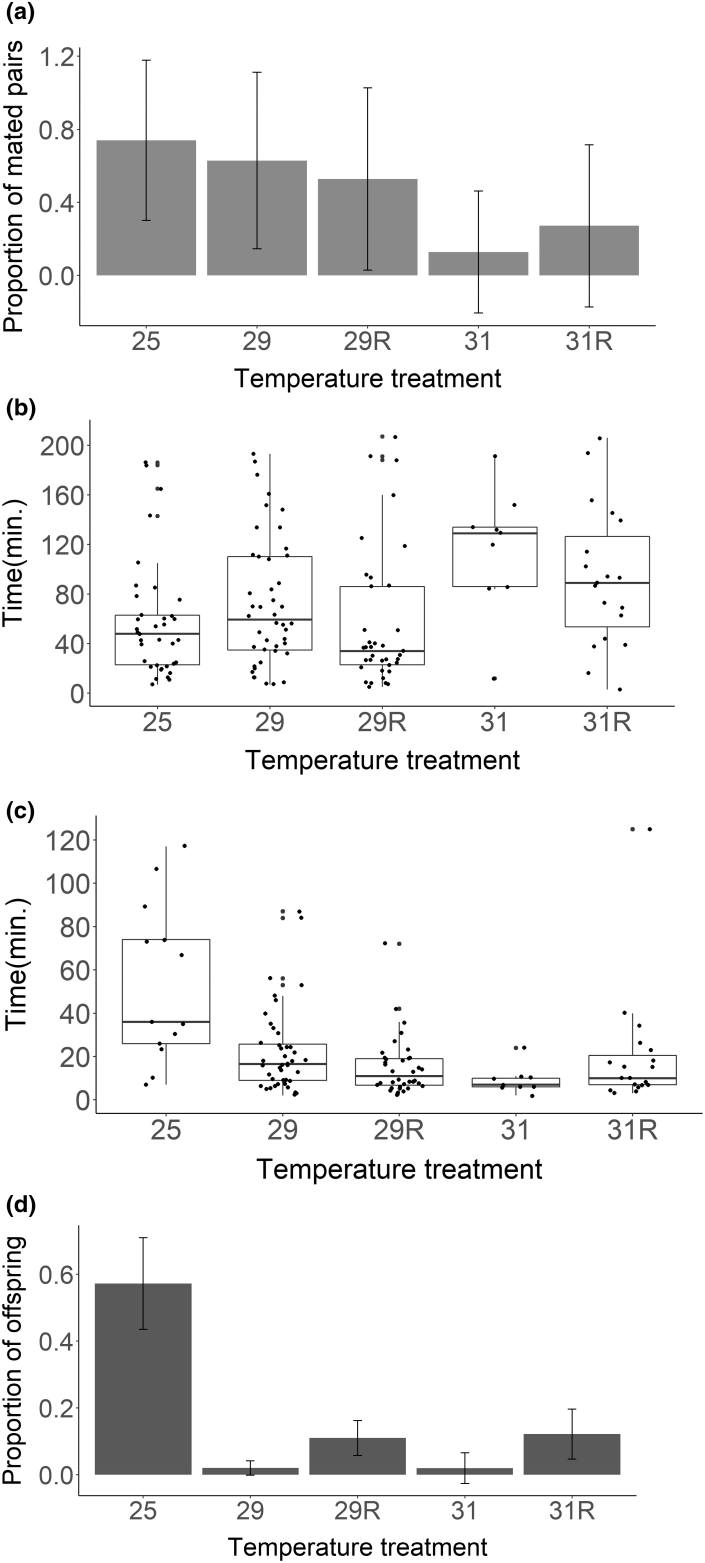
Male mating behavior and competitive success: Proportion of males gaining copulation (a) and their mating latencies (b). Mating latency of females remating with a competitor male (c), and proportion of offspring obtained by heat stressed males when defending their ejaculate against a second male (P1) (d). The results are shown according to the developmental temperature of first mating males. Males allowed to recover at 25°C after eclosion are represented with an “R”; otherwise, males were kept at the growth temperature after eclosion. Boxplots show in all cases the median with the highest and lowest data values (upper and lower lines).

When considering the mating behavior for individual pairs, we found little effect of moderate heat stress on mating success, and only males experiencing severe heat stress were negatively impaired. Males raised at 31°C gained few copulations compared to males from the other treatment groups (*χ* ^2^ = 66.135, *df* = 4, *p* < .0001; Figure 2a). Males raised at 31°C had longer mating latencies (GLM with gamma distribution: Deviance = 7.582, *F* = 3.002, *df* = 4, *p* = .021; Figure 2b) while there was no effect on copulation duration (GLM with Poisson distribution: Deviance = 8.867, *df* = 4, *p* = .065; Figure S2A).

In addition to single mating productivity, we also tested male sperm competitiveness after developmental heat exposure. We document a severe negative impact of heat on male sperm defense ability (GLM with a quasibinomial distribution: Deviance = 5173.8, *F* = 22.177, *df* = 4, *p* < .0001, Figure 2d), and even after we allowed males to recover for 5 days, only saw little improvement.

Additionally, heat‐stressed males were unable to prevent female remating, regardless of the possibility for recovery, as all females remated when the second male was present, while only 35.1% of females first mated to a control male remated (*χ* ^2^ = 82.624; *df* = 4, *p* < .0001). This pattern was also reflected in second mating latencies (GLM with Gamma error distribution; Deviance = 23.98, *F* = 7.04, *df* = 4, *p* < .0001; see Figure 2c).

### 3.2 Temperature effects on male reproductive tissues and mechanisms of recovery

#### 3.2.1 Sperm presence in the seminal vesicle and seminal vesicle size

**FIGURE 3:**
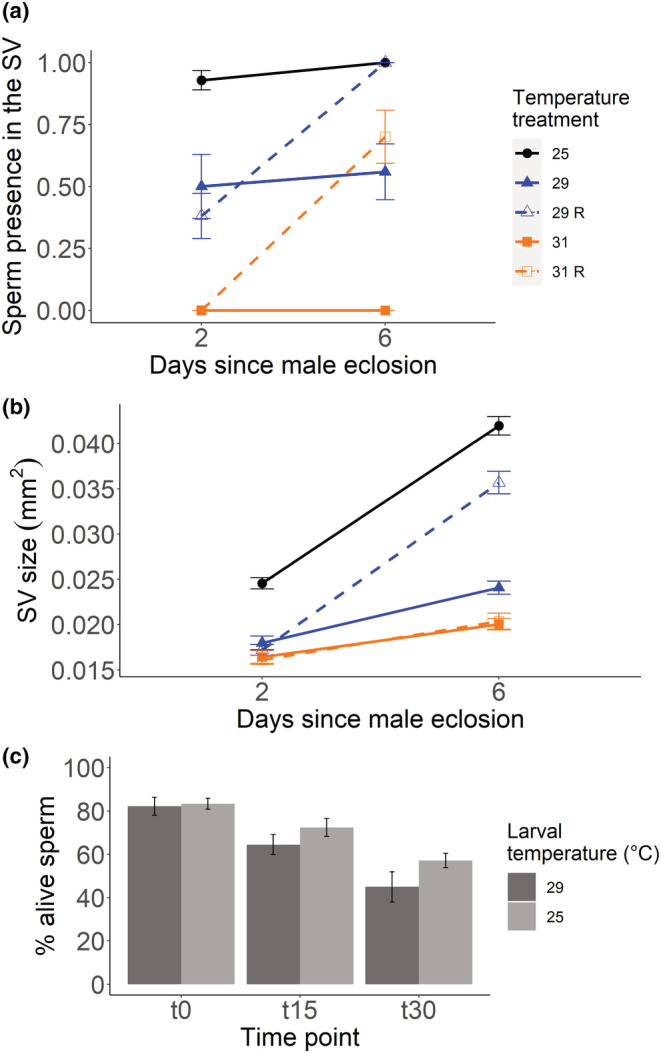
Assessment of mature sperm presence in heat‐stressed males (mean ± SE): Sperm presence in the seminal vesicles (SVs) (a) and vesicle size (b) of 2‐ and 6‐day‐old heat‐stressed males. Between 14 and 16 males were measured for each day and temperature treatment. For sperm presence, we recorded and assigned a score of 1‐ when sperm was present or 0‐ when the sperm was absent for each SV. When a male had one SV scoring 0‐ and one 1‐, then we overall assigned a score of 1‐ to that male. The mean of both SVs was used for each male for representing the SV size. Colored lines indicate the developmental temperature: 25 (circle symbol), black; 29 (triangle symbol), blue; or 31 (square symbol), orange. Males grown and kept after eclosion at the growth temperature are shown with a solid line while males allowed to recover (R) at 25°C after eclosion are shown with a dashed line. Sperm viability in heat‐stressed males (mean ± SE): Percentage of alive sperm for 6‐day‐old control and 29°C recovery males (c). Portrayed is the temporal decrease in sperm viability, measured at three different time points: just after the staining (t0) as well as 15 (t15) and 30 (t30) min later. Twenty‐one males from each temperature treatment were used.

We scored the presence of mature sperm in both seminal vesicles (SVs) and measured SV size in 2‐ and 6‐day‐old males as a proxy for the amount of mature sperm available to males. We found a major impact of elevated developmental temperatures and the opportunity to recover on the presence of mature sperm in the SV (*χ* ^2^ = 82.01, *df* = 4, *p* < .0001; Figure 3a). A 6‐day recovery resulted in a significant increase in mature sperm in the SVs for both temperature treatments while those males not allowed to recover did not improve even after 6 days.

The results we observed for sperm presence were paralleled by our measure of SV size (Figure 3b, Table 3). Overall, heat‐stressed males started with on average 31% smaller SVs than control males; however, by day 6, differences in SV size were more noticeable. Males raised at 29°C recovered to have only 15% smaller SVs compared to control males, while the other treatments retained small SV sizes resulting in a reduction of 52% for males developed at 31°C independently of the ability to recover and 43% for males raised and kept at 29°C.

**TABLE 3:** Results of a generalized linear model with a gamma error distribution for male accessory gland and seminal vesicle size. Both traits were measured in 2‐ and 6‐day‐old adult males.

Factor	Deviance	*F*	*df*	*p*
AG size				
Temperature	7.233	75.474	4	<.0001
Day	8.595	358.720	1	<.0001
Day^2^	4.736	197.670	1	<.0001
Wing length	0.531	22.144	1	<.0001
SV size				
Temperature	8.286	91.652	4	<.0001
Day	6.709	296.830	1	<.0001
Temperature × Day	0.804	11.437	4	<.0001

*Note*: Males previously developed at 25, 29, or 31°C and were kept at the growth temperature or moved to the control temperature to recover (R).

We also compared sperm viability in 6‐day‐old males from the control and the 29°C recovery treatment at three time points after releasing sperm from SVs (Figure 3c). This procedure allowed us to determine not only sperm quality, but also assess future sperm performance (Eckel et al., 2017). We found that, although the number of dead and alive sperm was similar at the beginning of the experiment (t0), sperm viability decreased faster over time (t15 and t30 min) when males were prior exposed to a moderate heat‐stress (GLMM with a binomial error distribution: temperature: *χ* ^2^ = 16.33, *df* = 1, *p* < .0001; time point: *χ* ^2^ = 24.22, *df* = 2, *p* < .0001; temperature × time point: *χ* ^2^ = 9.71, *df* = 2, *p* = .008).

#### 3.2.2 Accessory gland size changes during recovery

We here tested how developmental temperature affected accessory gland (AG) growth in young males. As developmental temperature affected male body size (GLM with a gamma error distribution: Deviance = 1.075, *df* = 4, *F* = 228.33, *p* < .0001) and this scales with organ size, we corrected for body size in further analyses using wing length as a proxy. Overall, males were smaller on day 6 after eclosion when they developed at higher temperatures (29: 1.25 mm ± 0.007; 29R: 1.23 mm ± 0.007; 31: 1.21 m ± 0.011; 31R: 1.17 mm ± 0.009) than control males (1.37 mm ± 0.007, see Table S2).

**FIGURE 4:**
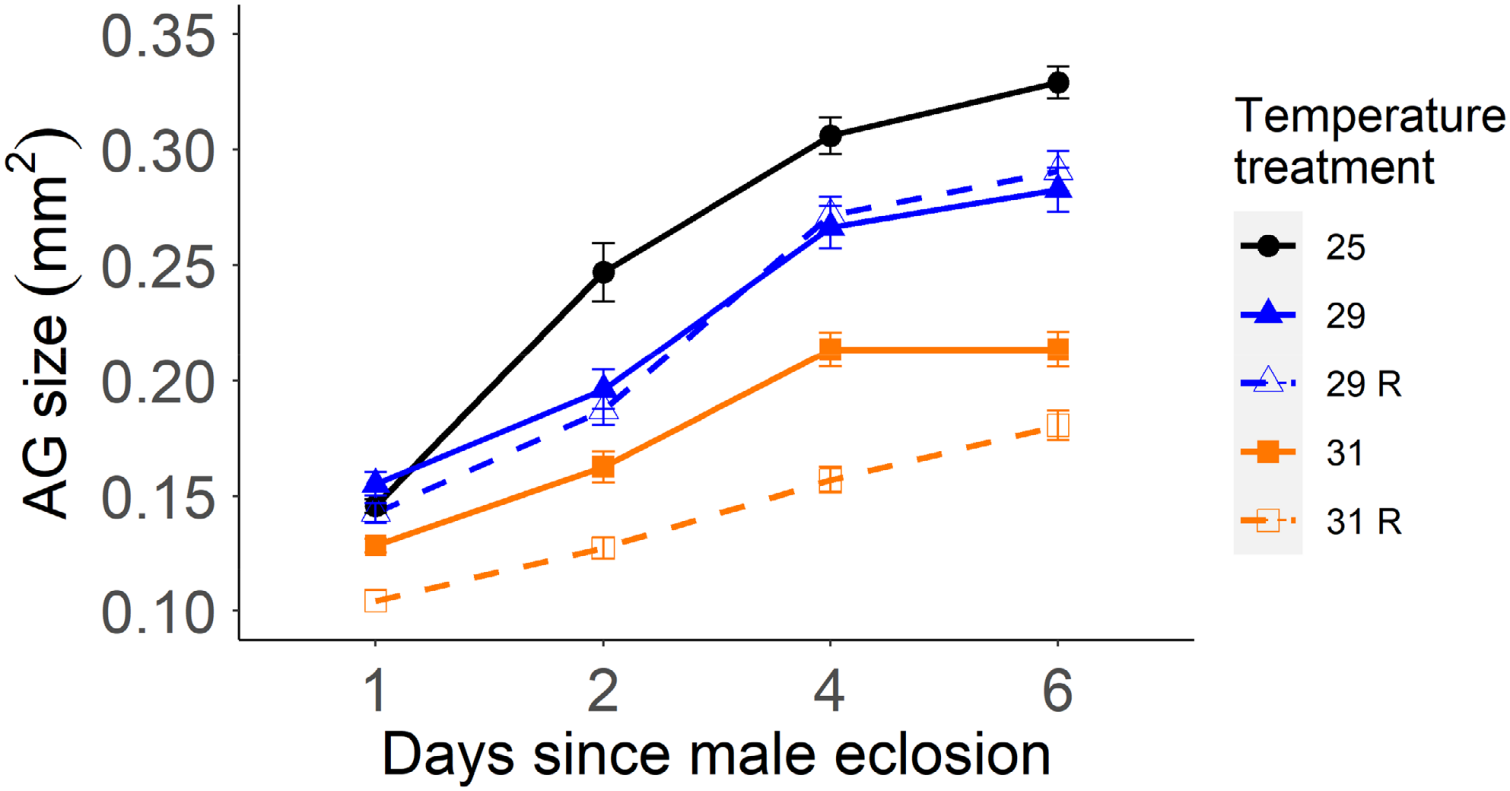
Sexual maturation of accessory glands in heat‐‐stressed males (mean ± SE): accessory gland size measures for 1‐, 2‐, 4‐, and 6‐day‐old heat‐stressed males. Between 15 and 26 males were measured for each day and temperature treatment. The mean of both accessory glands was used for each male. Colored lines indicate the developmental temperature (°C): 25 (circle symbol), black; 29 (triangle symbol), blue; or 31 (square symbol), orange. Males grown and kept after eclosion at the growth temperature are shown with a solid line while males allowed to recover (R) at 25°C are shown with a dashed line.

We observed a clear reduction in AG growth during the first 6 days after eclosion in males exposed to elevated developmental temperatures (Figure 4, Table 3). The reduction in growth was gradual: the higher the temperature, the slower the increase in AG size, resulting in significant differences on day 6. Surprisingly, AG growth for males who developed at 29°C did not depend on the possibility to recover or not (see Figure 4 and post hoc tests in Table S3). Interestingly, the AG sizes of recently eclosed males (day 1) were fairly similar. In a second analysis, we only compared the AG size of heat‐stressed males and hence, excluded the control group from the analysis, using larval temperature and the possibility of recovery or not as fixed variables. This revealed the significant effect of the ability to recover (GLM with a gamma error distribution, recovery: *F* = 18.88, *df* = 1, *p* < .0001), with the subsequent post hoc test (Table S3), implying that this was mainly driven by males in the 31°C recovery treatment having significantly smaller AGs compared to males not allowed to recover. By day 6, the reduction in size was 12.8% for males of both treatments exposed to 29°C and 40.3% for those exposed to 31°C compared with the control group.

We next assessed whether the allometric relationship between the gland and body size was also altered. The regression analysis between both factors (see Table S4 and Figure S3) indicates that in the two elevated temperature treatments, a hypometrical relationship prevails (*b* < 1) highlighting that the gland is smaller than expected for the body size, while it tends to be isometrical in males grown at 25°C (*b* = 1). Thus, differences in AG size can be attributed to altered growth patterns due to temperature. Overall, these results suggest that AG maturation is inhibited by elevated temperatures with males ultimately having smaller glands.

## 4 DISCUSSION

We here investigated the recovery dynamics of a temperate *D. melanogaster* strain with a special focus on effects on male reproductive tissues. Overall, sub‐lethal temperatures severely affected a male's ability to reproduce as found in other ectotherms (Conrad et al., 2017; Nguyen et al., 2013; Parratt et al., 2021; Rodrigues et al., 2021, 2022; Sales et al., 2018, 2021; Vasudeva et al., 2019; Walsh et al., 2019; Zheng et al., 2017; Zwoinska et al., 2020). In accordance with previous findings (Chakir et al., 2002; Petavy et al., 2001), the vast majority of males (60%–100%) were temporarily sterile during the first days after eclosion when exposed to temperatures above 29°C during development and then often regained fertility. Moreover, in young males recovering from immediate heat stress, we found negative effects on most of our measured reproductive traits that can be explained by males transferring a sub‐optimal ejaculate. Despite the ability to recover for 6 days, we found temperature stress to still lead to severe fitness reductions with recovery dynamics depending on the developmental temperature experienced. This observed pattern is likely due to slowed growth of the accessory glands (AG) and alteration of sperm production, which could explain the inability of males to fully recover fertility levels within our assay period.

The observed reduction in reproductive output could not be explained by reduced male mating rates, that is, due to males becoming unattractive as found in male red mason bees (*Osmia bicornis*, Conrad et al., 2017), or *D. pseudoobscura* (Sutter et al., 2019), as we found little effect of a moderate heat stress of 4° over the optimal temperature on mating success and behavior. We rather suspected males were not able to produce and/or transfer an adequate ejaculate. Developmental temperature can result in aberrant sperm in *D. melanogaster* (Rohmer et al., 2004) potentially explaining our reduced egg‐to‐adult survival. Even if males can produce sperm, they might transfer less, reducing their overall fertility (Kraaijeveld & Chapman, 2004; Seo et al., 1990; Taylor et al., 2001). Males of the parasitoid wasp *Anisopteromalus calandrae* failed to produce mature sperm even after 10 days of recovery from a developmental heat shock, and as a result, females stored less sperm and were less fertile (Nguyen et al., 2013). Constant exposure to heat stress during development resulted in reduced testes and sperm size in the bruchid beetle *Callosobruchus maculatus*, but no reduction in fertility after a single mating was found (Vasudeva et al., 2014). Females could discriminate against low‐quality ejaculates, for example, by ejecting those ejaculates as found in *D. melanogaster* (Lüpold et al., 2013), or increase their willingness to remate as found here and in *D. pseudoobscura* (Sutter et al., 2019). This facultative polyandry could be a behavioral strategy to promptly ensure the gain of a fertile ejaculate to mitigate potential fitness losses from heat‐induced sterility of her first mate.

In addition to single‐mating productivity, we also tested male sperm competitive ability after developmental heat exposure. Competitive ability is key to male reproductive success (Simmons, 2001) and was sensitive to thermal conditions in *T. castaneum* (Sales et al., 2018). We found a similar negative impact of heat on male sperm competitiveness even after we allowed males to recover for 5 days. Thus, overall, we also observe the previously described sensitivity of male reproductive function to elevated, but sub‐lethal temperatures (Chakir et al., 2002; David et al., 2005; Sales et al., 2018; Walsh et al., 2019) in both competitive and non‐competitive contexts. Reduced ability to fertilize eggs and win during sperm competition could be due to reduced sperm transfer (Kraaijeveld & Chapman, 2004; Seo et al., 1990; Taylor et al., 2001) and/or reduced sperm storage by females (Nguyen et al., 2013). Both traits are important determinants in *D. melanogaster* sperm competition outcomes (Lüpold et al., 2013; Manier et al., 2010). However, sperm storage and sperm competitive ability are also mediated by the receipt of SFPs, secreted by the AGs (Avila et al., 2011). Thus, we continued by looking at the SVs as a proxy for sperm maturation and the AGs for possible heat damage and we will discuss those two in turn.

Within 6 days, males can recover fertility reaching up to 60% of control male productivity, indicating that spermatogenesis was not completely damaged. Both spermatogenesis and juvenile development last 10 days at 25°C in *D. melanogaster* (Demarco et al., 2014; Fabian & Brill, 2012), whereby normally, about 3 days after oviposition, third instar larvae start to develop testes and hence, for spermatogenesis to start. The first sperm is produced at the early pupal stage (prepupa), and under normal conditions, it starts the maturation phase (Fabian & Brill, 2012). Thus, males as young as 1 day post‐eclosion can successfully fertilize eggs (Ruhmann et al., 2016). Sperm continuously accumulates during early adulthood into the SVs encompassing the time when males are approximately 2 days old (when males of this species become sexually mature) until about 5 days old. Thus, our time window covers the normally critical period of sexual maturation; however, in our heat challenge treatments we could have likely seen further improvement beyond the assay period if we had observed males for at least one spermatogenesis cycle for recovery, for example, as done in *D. virilis* (Walsh et al., 2021) or *T. castaneum* (Sales et al., 2021). In our assays, heat‐stressed males started producing offspring by day 4 of the recovery process with the exception of males that had developed at 31°C, which needed much longer to recover fertility. As sperm individualization is temperature sensitive (Ben‐David et al., 2015), high developmental temperatures might disrupt proper sperm maturation, as found in *D. suzukii* (Kirk Green et al., 2019). As the last step, the 64 interconnected spermatids individualize, and finally, the mature sperm coils into the base of the testis (Fabian & Brill, 2012; Steinhauer, 2015), and already at 29°C, Ben‐David et al. (2015) observed the formation of fewer and more abnormal individualization complexes. Our proxy for the availability of mature sperm—SV size and sperm presence in the SVs—not only corroborated these findings with a significant impact of elevated developmental temperatures and the opportunity to recover on the presence of mature sperm in the SVs but also highlighted a delay in mature sperm formation. Although we found that sperm presence in the SVs of males allowed to recover improved over time, sperm quantity was lower than in control males. Apart from having fewer sperm, this sperm also seems more sensitive, as sperm viability decreased faster in males exposed to 29°C during development within 30 min after collection with the possibility to lead to reduced fertilization success in the long run. A reduction in the number of sperm ejaculated was also found in *T. castaneum* males when facing a heat shock of 7°C above the optimum temperature (Sales et al., 2018). This reduction might be the result of a significant increase in sperm cell death of exposed males, as we found with time. While our data show that recovery of spermatogenesis is possible, the effects of temperature are not completely compensated during our 6‐day recovery. However, if we had given males longer, a full recovery might have occurred as was observed in *T. castaneum* (Sales et al., 2021). We additionally show, when elevated temperatures persist and recovery is not allowed, sperm maturation and/or movement into the SV is not possible, as no sperm was found in the SVs of 6‐day‐old males grown and kept at 31°C. A result was similarly found in *D. suzukii* males raised at 30°C (Kirk Green et al., 2019) and in line with the idea that spermiogenesis is affected as found previously (Ben‐David et al., 2015) halting the maturation of sperm. However, there is the potential for strong variation across genotypes in their ability to produce mature sperm as indicated by the variation in fertility at sub‐lethal temperatures across isogenic lines of the *Drosophila* Genetic Reference Panel (Rodrigues et al., 2021).

In addition to the SVs, we also investigated the response to elevated developmental temperature on accessory gland maturation as it is the main production site of SFPs, which are important determinants of male reproductive success (Avila et al., 2011; Chapman et al., 2003). The interplay among male SFPs, sperm, and the female reproductive tract is integral to ensure that all stages of the reproductive cascade can proceed and culminate in the fertilization of a passing ova (Avila et al., 2011). Additionally, male SFPs can protect sperm and enhance sperm viability (den Boer et al., 2008, 2009; Holman, 2009b; King et al., 2011). The growth of the accessory gland is key during sexual maturation (Ruhmann et al., 2016) and is accompanied by an increase in functionality (Leiblich et al., 2012; Prince et al., 2019). We here observed a negative impact of heat stress during development with a clear reduction in AG growth during the early stages. Surprisingly, recovery had little effect and did not aid AG maturation, which could result in reduced AG functionality, affecting SFP properties and/or composition. This hypothesis is tentatively supported by our phenotypic data, as heat‐stressed males were not able to prevent female remating, induce increased oviposition (a trait mediated by ejaculatory sex peptide [Chapman et al., 2003; Liu & Kubli, 2003] and ovulin [Rubinstein & Wolfner, 2013]), and defend their ejaculate against subsequent rivals, regardless of the possibility for recovery. These traits are determined by seminal fluid proteins like the sex peptide (Avila et al., 2010; Chapman et al., 2003; Fricke et al., 2009; Liu & Kubli, 2003) and our results point toward the possibility that heat‐stressed males could not transfer functional or adequate amounts of sex peptide and potentially other SFPs. We worked under the premise that a larger AG size is indicative of SFP accumulation, which is an adequate proxy for at least the first 3 days after eclosion (Koppik et al., 2018).

Under normal circumstances, the rapid growth of the AG can be observed in the first 10 days after eclosion (Box et al., 2019; Ruhmann et al., 2016) and continues at a lower rate during male adulthood (Box et al., 2019). In general, the change in AG size occurs due to changes in both its cell types—the secondary and the main cells (Box et al., 2019; Leiblich et al., 2012). Main cells increase in size due to endocycling throughout male life (Box et al., 2019). In secondary cells, the vacuole‐like compartments (VLCs) increase in size and change in nature and location (Prince et al., 2019). VLCs are vital to secondary cell functionality (Corrigan et al., 2014; Gligorov et al., 2013; Prince et al., 2019) by secreting their content into the gland lumen and communicating with neighboring main cells (Hopkins et al., 2019). Secondary cell and proper VLC maturation are important for overall AG functionality and thus the question arises, whether development at elevated temperatures disrupts the proper formation of secondary cells and/or interferes with main cell growth. As we did not observe an improvement for males allowed to recover compared with males kept at the growth temperature, this might indicate that processes involved in AG growth cannot be rescued and developmental temperatures might produce an irreversible damage, which could explain the inability of heat‐damaged males to recover and reach the fitness of control males. However, the AG can regenerate from damage (Box et al., 2019), and possibly our chosen time span was too short to see this effect after heat damage, warranting further investigations.

## 5 CONCLUSION

Under predicted climate change scenarios, an increase in temperature is expected next to an occurrence of longer and more severe heat waves (Collins et al., 2020; Meehl & Tebaldi, 2004). Temperatures in the range of 4–6° above the optimum temperature as tested here are easily reachable in many areas worldwide, especially during the summer months (Solomon et al., 2007). From an ecological point of view, this could lead to severe consequences for species distributions and persistence, particularly as recent research highlights a lack of genetic variability in male sub‐lethal fertility limits (van Heerwaarden & Sgrò, 2021; Zwoinska et al., 2020). This lack would severely hamper a specie's ability to mitigate escape from this predicament through evolutionary adaptation to the novel conditions or to exhibit some levels of phenotypic plasticity. Already now, a specie's thermal fertility limit is a better predictor of species ranges than the critical thermal limit across 43 *Drosophila* species (Parratt et al., 2021). Our findings echo this recent interest in understanding the impact of temperature on male reproduction. We here add insights on the fitness costs of heat stress and mechanisms allowing recovery. In sum, we show that sub‐lethal thermal sterility and the subsequent fertility reduction during recovery could be caused by a combination of malfunctioning reproductive traits: inefficient functionality of the accessory glands and alteration of spermatogenesis. In addition, we show that the possibility of recovery after exposure, even when facing a moderate heat stress, does not mitigate the damage imposed on early adulthood reproduction induced by elevated thermal stress during development. Moreover, 5 days of recovery is not enough to rescue SFP functionality, and the AGs fate is mainly determined during development, which could explain the inability of heat‐damaged males to recover and reach the fitness of control males. We found AG functionality more thermosensitive than spermatogenesis as SFPs‐induced female post‐mating responses were already impaired at a moderately stressful temperature of 4° over the control temperature. Mature sperm though was found in males raised at 29°C and particularly in those allowed to recover, which could explain the progressive increase in fertility observed in recovering males.

## AUTHOR CONTRIBUTIONS

**Berta Canal Domenech:** Data curation (lead); formal analysis (lead); investigation (equal); methodology (lead); resources (equal); validation (equal); visualization (lead); writing – original draft (lead); writing – review and editing (equal). **Claudia Fricke:** Conceptualization (equal); data curation (supporting); formal analysis (equal); funding acquisition (lead); investigation (equal); methodology (supporting); project administration (lead); resources (equal); supervision (lead); validation (lead); visualization (supporting); writing – original draft (supporting); writing – review and editing (equal).

## CONFLICT OF INTEREST

We declare to have no competing interests.

## Supporting information


Appendix S1

## Data Availability

Data has been updated successfully in Dryad. DOI: https://doi.org/10.5061/dryad.k6djh9w9w.
