# Supplementary material for: Recovery from heat‐induced infertility—A study of reproductive tissue responses and fitness consequences in male Drosophila melanogaster
Source: Ecol Evol. 2022 Nov 30;12(12):e9563. doi: 10.1002/ece3.9563 (PMC9712812; doi:10.1002/ece3.9563)

**Appendix manuscript**

Recovery from heat-induced infertility – a study of reproductive tissue responses and fitness consequences in male *Drosophila melanogaster*

**Additional tables**

**Table S1**. Proportion of fertile males and offspring number produced on day 1, 2, 4, or 6 after eclosion. A generalized linear model following a binomial distribution was used to analyze the proportion of fertile males. A Zero-inflated negative binomial model was used for the offspring number. The model included a binomial part that analyzed whether an offspring was produced or not, while the count part analysed the number of offspring produced following a negative binomial distribution. Males previously developed at 25, 29 or 31°C and were kept at the growth temperature or moved to the control temperature to recover. In all cases, male developmental temperature and opportunity to recover were analysed together as one factor (temperature) with five levels, with day of measurement coded as a factor with four levels. These results are complementary to the fecundity experiment (sub-lethal temperature effects on behavior and male reproductive output). Offspring number measurements were repeated, showing a similar pattern as found in table 1 in the main text.

| ***Proportion of fertile males*** | | | |
| --- | --- | --- | --- |
|  | ***df*** | ***Deviance*** | ***P*** |
| **Temperature * Day** | 12 | 101.32 | < 0.0001 |
| **Temperature** | 4 | 315.4 | < 0.0001 |
| **Day** | 3 | 58.27 | < 0.0001 |
| ***Offspring number*** | | | |
|  | ***df*** | ***Chi square*** | ***P*** |
| **Binomial part** | | | |
| **Temperature * Day** | 12 | 100.46 | < 0.0001 |
| **Count part** | | | |
| **Temperature** | 4 | 15.49 | 0.004 |
| **Day** | 3 | 7.70 | 0.053 |

**Table S2**. Post-hoc test comparison of wing length on day six after male eclosion for data collected as part of the ‘*accessory gland size changes during recovery’* subsection in the main text. Temperature treatment was used as a fixed factor again combining all five treatments of males kept at the growth temperature as adults or moved to the control temperature for recovery (R).

| **Temperature (°C)** | ***Estimate*** | ***SE*** | ***z-value*** | ***P*** |
| --- | --- | --- | --- | --- |
| **25 – 29R** | 0.079 | 0.004 | 19.530 | < 0.001 |
| **25 – 29** | 0.079 | 0.004 | 19.341 | < 0.001 |
| **25 – 31R** | 0.116 | 0.004 | 26.987 | < 0.001 |
| **25 – 31** | 0.103 | 0.004 | 23.066 | < 0.001 |
| **29 – 29R** | -0.0002 | 0.004 | -0.052 | 1.000 |
| **31 – 31R** | 0.013 | 0.004 | 2.625 | 0.066 |

**Table S3**. Results of pairwise comparisons using t-tests comparing data for male accessory gland size on day 6 for males grown at 25°C and males grown at 29 or 31°C and kept at the growth temperature after eclosion or moved to the control temperature to recover (R); see table 2 in the main text for the main analysis. Temperature treatment was used as fixed factor with five levels combining the different temperature treatments.

| **Contrast** | ***P*** |
| --- | --- |
| **25 – 29** | 0.0015 |
| **25 – 29R** | 0.0013 |
| **25 – 31** | < 0.0001 |
| **25 – 31R** | < 0.0001 |
| **29 – 29R** | 0.993 |
| **31 – 31R** | 0.002 |

**Table S4**. Results of a regression analysis between accessory gland (AG) size and wing length to assess allometry between both organs as described in the ‘*accessory gland size changes during recovery’* subsection in the main text. Males were grown at 25, 29 or 31°C and kept at the growth temperature after eclosion or moved to the control temperature to recover (R). Both AG size and wing length measurements were converted into the same units (µm^2^) and data were log transformed to calculate intercepts and slopes. Day was included in the model as a fixed factor.

| **Temperature (°C)** | ***term*** | ***estimate*** | ***std.error*** | ***statistic*** | ***P*** |
| --- | --- | --- | --- | --- | --- |
| 25 | (Intercept) | -3.657 | 2.593 | -1.41 | 0.162 |
| 25 | log(Wing mean) | 1.076 | 0.179 | 5.994 | <0.0001 |
| 29 | (Intercept) | -0.152 | 3.726 | -0.04 | 0.966 |
| 29 | log(Wing mean) | 0.848 | 0.261 | 3.246 | 0.001 |
| 29R | (Intercept) | 6.887 | 3.301 | 2.086 | 0.04 |
| 29R | log(Wing mean) | 0.349 | 0.232 | 1.505 | 0.136 |
| 31 | (Intercept) | 3.923 | 3.561 | 1.102 | 0.275 |
| 31 | log(Wing mean) | 0.551 | 0.25 | 2.2 | 0.032 |
| 31R | (Intercept) | 10.047 | 4.58 | 2.194 | 0.031 |
| 31R | log(Wingmean) | 0.105 | 0.324 | 0.325 | 0.746 |

**Additional figures**

**Fig. S1** Proportion of fertile males (A) measured as the fraction of males at least producing one offspring and (B) number of offspring after a single mating; mean ± SE. Males were allowed to mate for 24h when one-, two-, four- or six- days old after eclosion. Males previously developed at (°C): 25 (circle symbol, black line); 29 (triangle symbol, blue line); or 31 (square symbol, orange line). Males grown and kept after eclosion at the developmental temperature are shown as a solid line, while males allowed to recover (R) at 25°C after eclosion, are shown as a dashed line. Boxplots show in all cases the median with the highest and lowest data values (upper and lower lines). Table S2 above shows the statistical results for the offspring number.

**
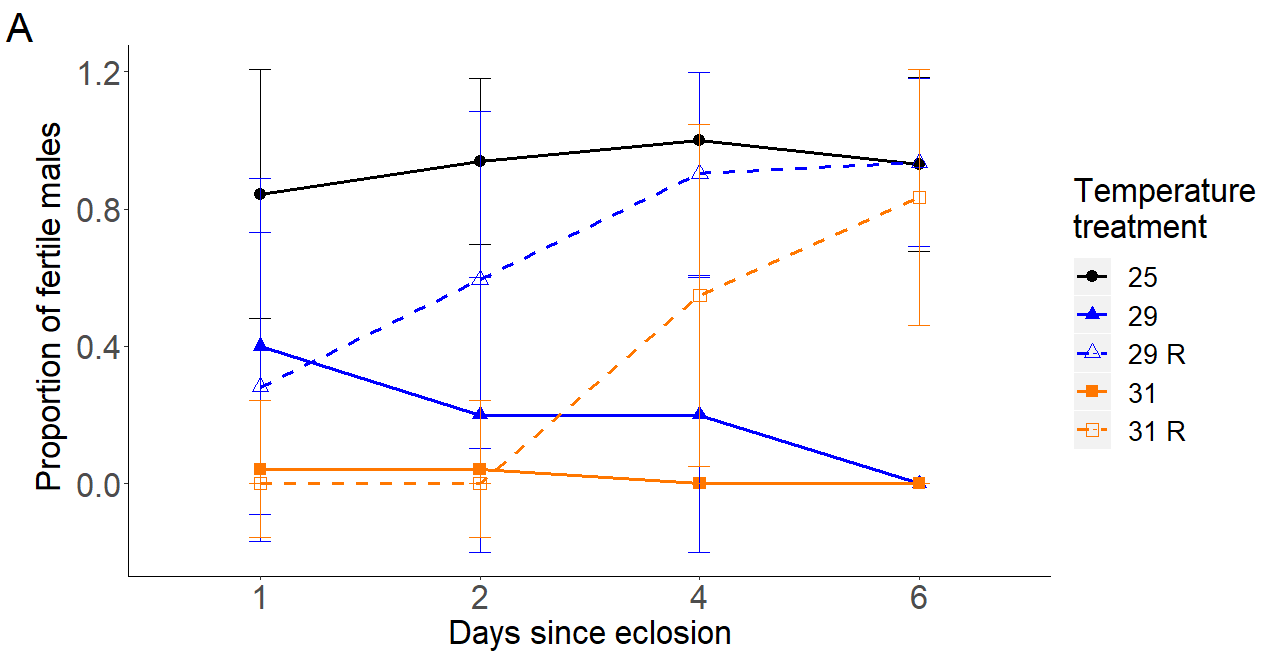
**


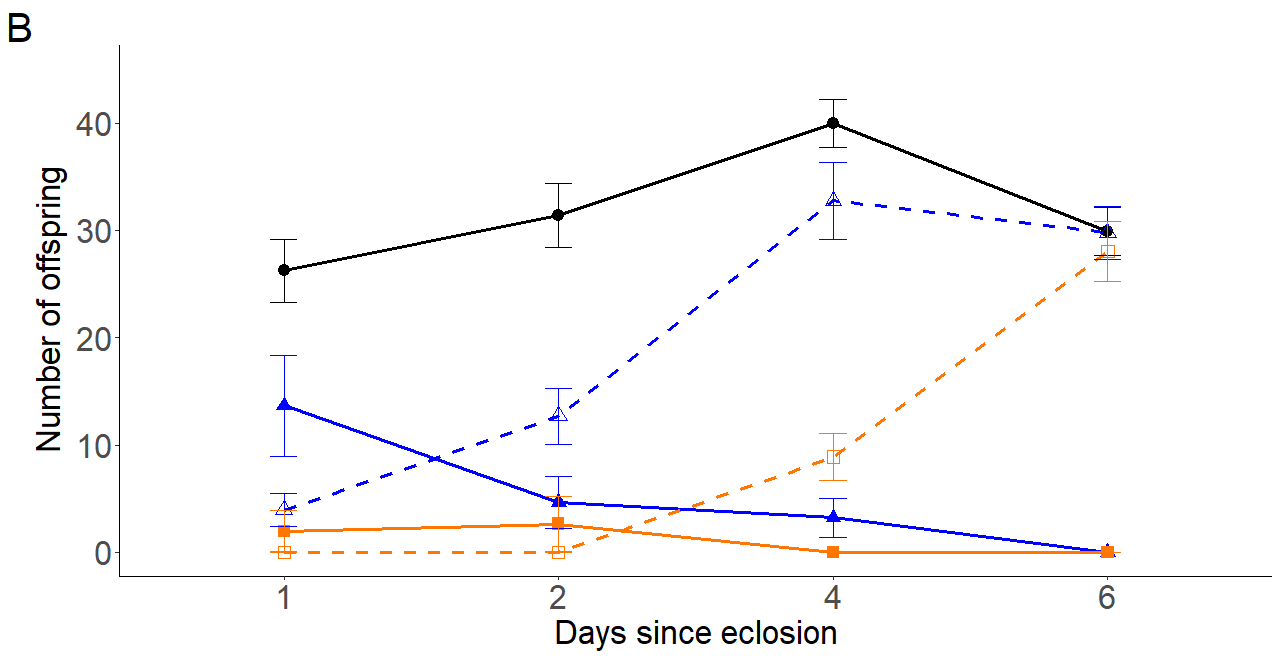


**Fig. S2** **Duration of copulation in heat-challenged males:** duration of copulation (A) when encountering a single virgin female in a no-choice setting and duration of copulation (B) of females remating with a competitor male 2 hours after the first mating. Data was collected as part of the sperm competition assay. The results are shown according to the developmental temperature of first mating males. Males allowed to recover at 25°C after eclosion are represented with an “R”; otherwise, males were kept at the growth temperature after eclosion. Boxplots show in all cases the median with the highest and lowest data values (upper and lower lines).

**
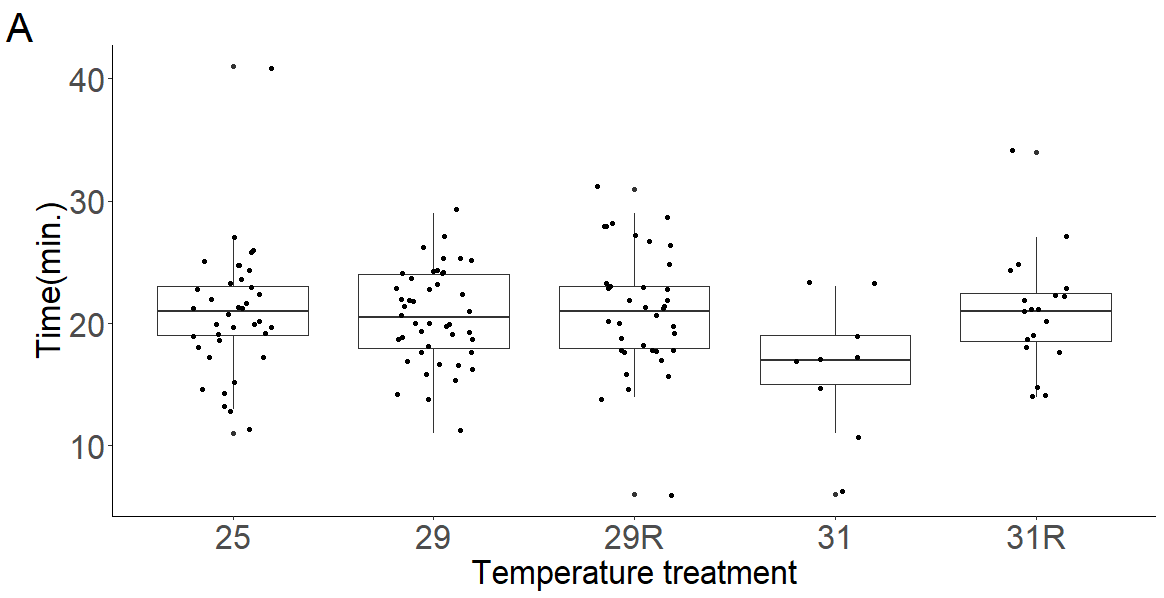
**

**
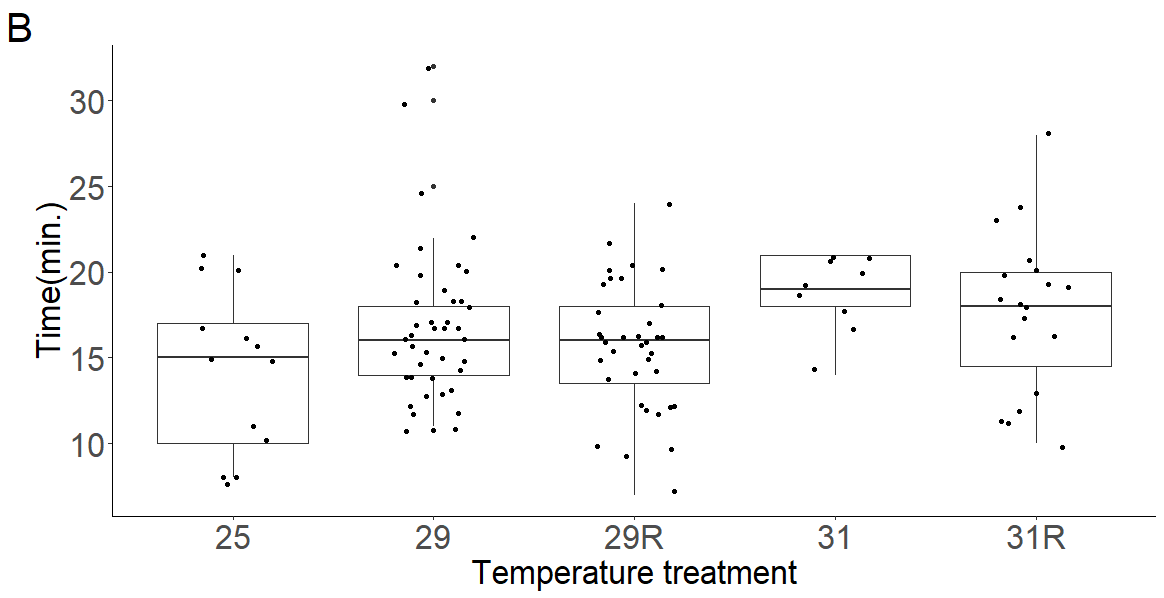
**

**Fig S3.** Ontogenetic allometries of accessory gland (AG) area against wing length on day 1 (A), 2 (B), 4 (C) and 6 (D) after eclosion. Males from the different temperature treatments are indicated as follows: 25°C, black; grown and kept at 29°C after eclosion, dark blue; grown at 29°C allowed to recover, pink; grown and kept at 31°C after eclosion, orange; and grown at 31°C allowed to recover, light blue. Data was log-transformed and converted into the same units (mm^2^).


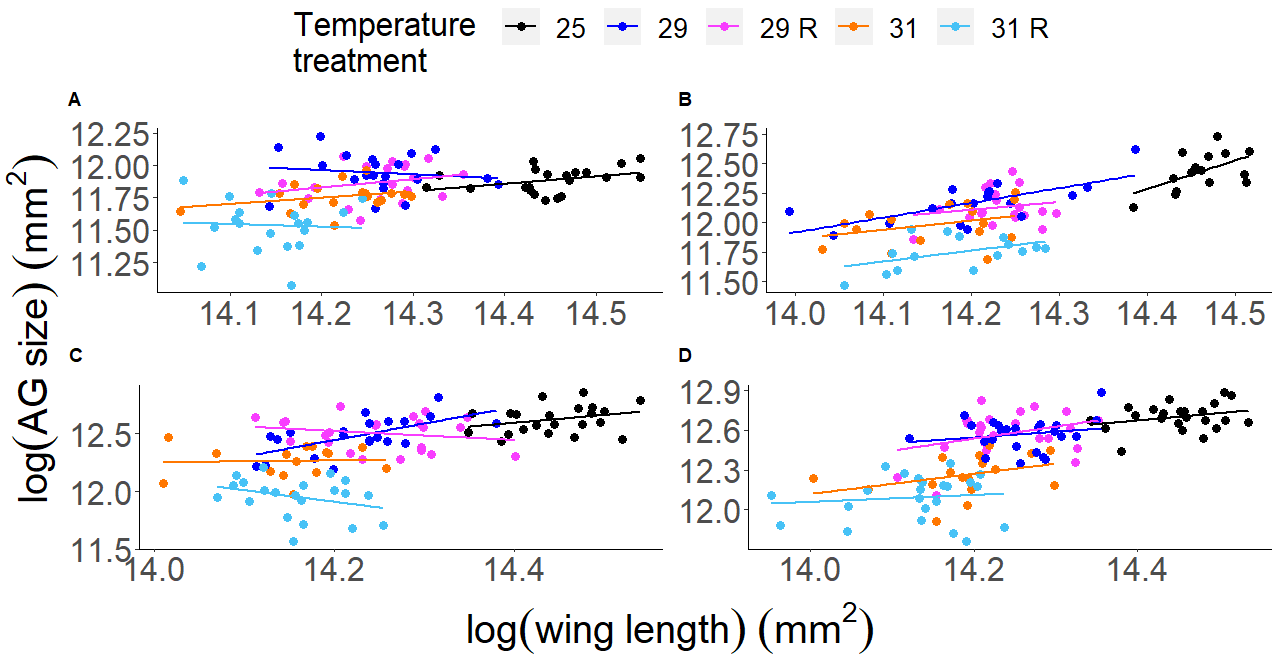

Supplement: Supplementary file 1 — Appendix S1 [file ECE3-12-e9563-s001.docx]
